# Flow Synthesis of Biologically-Relevant Compound Libraries

**DOI:** 10.3390/molecules25040909

**Published:** 2020-02-18

**Authors:** Pilar María Luque Navarro, Daniela Lanari

**Affiliations:** Dipartimento di Scienze Farmaceutiche, Università degli Studi di Perugia, 06123 Perugia, Italy; pilarmaria.luquenavarro@studenti.unipg.it

**Keywords:** flow chemistry, compound library, bioactive molecules

## Abstract

Flow chemistry is one of the most prominent enabling technologies that has greatly shaped the way chemists’ approach organic synthesis. Specifically, in drug discovery, the advantages of flow techniques over batch procedures allow the rapid and efficient assembly of compound libraries to be tested for biological properties. The aim of the present review is to comment on some representative examples from the last five years of literature that highlight how flow procedures are becoming of increasing importance for the synthesis of biologically-relevant molecules.

## 1. Introduction

In the past decade, enabling technologies, i.e., all the methodologies that can speed-up chemical processes as well as simplify work-up procedures have greatly influenced the approach of chemists toward the development of new synthetic protocols [1,2,3,4]. In the broad landscape of enabling technologies in medicinal chemistry, flow chemistry stands out as one of the most powerful techniques to discover new active pharmaceutical ingredients or to set up a streamlined process for their manufacturing. A plethora of articles and reviews well-documented how many chemical processes could benefit from running reactions in flow [5,6,7,8,9]. In particular, this approach has demonstrated to be superior to batch protocols in every case where heat transfer and mixing are important, hazardous or sensitive materials are handled, and high temperatures/pressures are required. Furthermore, this technology is considered a green synthetic approach as it often delivers better product yields than those derived from batch protocols, and also it allows easier product purification leading to minimum waste production [10,11,12]. In the drug discovery field, the need for novel molecules with drug-like structures to be used for high-throughput screening required enabling technologies for their production; in this context, flow chemistry represents the ideal tool for generating compound libraries in a fast and automated fashion [13]. This short review introduces some examples from the last five years on the use of flow chemistry for the assembly of pharmaceutically relevant compound libraries. The advantages of the flow protocols over batch procedures are discussed. The selected papers described flow procedures employing different types of equipment, either commercial or home-made, and different types of reactors (chip, coil, and packed bed reactors). This choice has been made to highlight how flow chemistry has reached a mature age and, therefore, can be reliably employed to perform reactions in a wide variety of conditions overcoming experimental difficulties that could make some processes impossible to occur or with a very low yield in batch conditions.

## 2. *N*,*O*-Based Compound Libraries

**Scheme 1:**
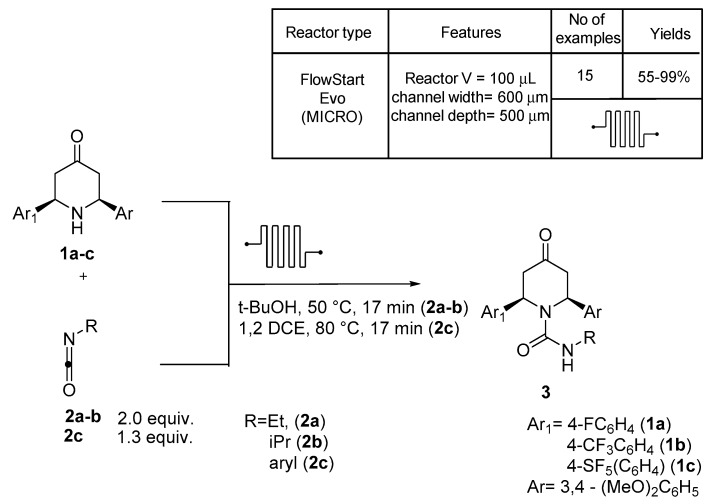
Flow synthesis of the urea-containing compound library from the piperidin-4-one moiety.

Urea moiety is of fundamental importance in pharmaceutical and agrochemical sciences thanks to its ability to interact through hydrogen bonding with biomolecules. In the literature, a wide variety of urea-based biologically active compounds are present like vestipitant and trimefluor [14,15]. As a continuation of previous work [16], Rutjes and co-workers set up a new procedure for the synthesis in flow of a library made of urea-based molecules based on piperidin-4-ones (Scheme 1) [17]. Optimization studies have been performed using as test compounds ethylisocianate **2a** and piperidin-4-one **1a** employing a microreactor (100 µL effective volume). After using different solvents, temperatures, and reactants ratios, the authors identified as best reaction conditions those that employed tBuOH as solvent at 50 °C for 17 min. On the other hand, when isocyanate **2c** bearing an aryl moiety was allowed to react with **1a**, full conversion was obtained only by using 1,2 dichloroethane (1,2 DCE) at 80 °C for 17 min. Such conditions were applied then to isocyanates (**2a**–**c**) and piperin-4-ones (**1a**–**c**) for the synthesis of 15 urea-based products **3** containing different fluorine moieties as it has been shown that such functionalities could enhance bioactivity and metabolic profile [18]. The urea-based products were obtained with isolated yields values ranging from 55 to 99%. No further chromatography purification was necessary. This methodology, therefore, is suitable for the synthesis of a large number of molecules to be tested for biological activity.

**Scheme 2:**
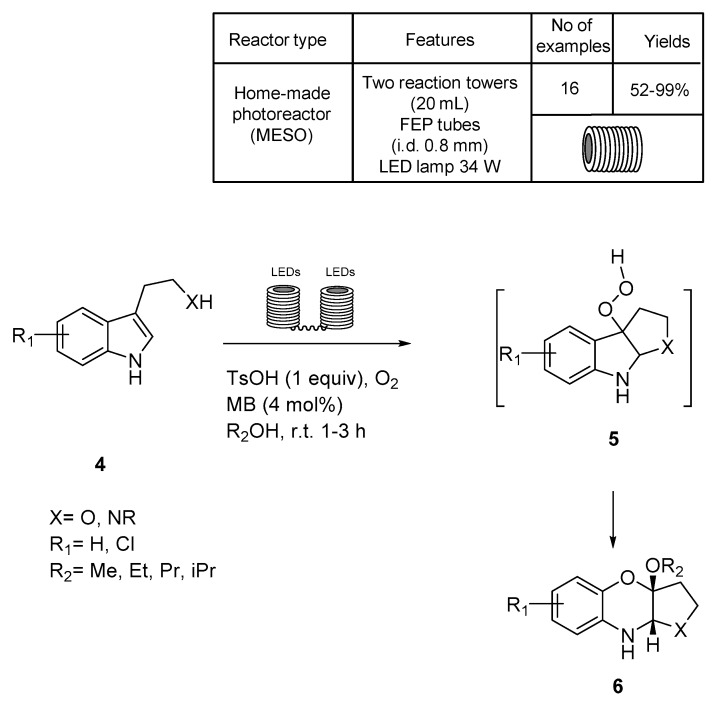
Continuous flow synthesis of tricyclo-1,4-benzoxazines **6** catalyzed by visible-light.

1,4 Benzoxazines are ubiquitously found in pharmaceutically active compounds as they can exhibit neuroprotective, anti-inflammatory, antibacterial properties, among others [19,20]. Chen and co-workers have developed a new strategy in flow for the synthesis of tricyclo-1,4-benzoxazines through intramolecular cyclization of indole derivatives (Scheme 2) [21]. Previous studies from the same research group showed that batch reaction suffered from many drawbacks as limited scope, difficulties in scaling up, and low reaction temperature requirement (−70 °C).

Optimization of the reaction in flow has been performed using a home-made capillary continuous-flow photoreactor already used in another work from the same authors [22]. It was assembled by connecting in series two reactors towers made of quartz glass tubes (i.d. 75 mm) with a fluorinated ethylene propylene FEP tube coiled inside and irradiated by an LEDs corn light bulb (34 W). The indole derivative **4** in the presence of catalytic methylene blue (MB), O_2_, and alcohol first yielded the peroxide intermediate **5**, which after a rearrangement, gave the desired tricyclic-1,4-benzoxazine **6** at room temperature. The best acid to be employed resulted in being TsOH; while the reaction didn’t work in the absence of O_2_, yields were lower when CH_3_CN was used as the solvent instead of alcohol. The scope of reaction was tested by synthesizing a library of 16 products using methanol, ethanol, propanol, and isopropanol as alcohol. All the products have been obtained in 1–3 h, whilst the batch procedure required 24 h. The gram-scale reaction was also performed to prove the scalability of the new synthetic protocol.

An innovative flow-procedure for the synthesis of 2-aminophenoxazin-3-ones has been proposed by Lanari and co-workers [23]. Such molecules are of interest for their use as antibiotics like questiomycin A, so a new methodology for their production is indeed of great interest [24].

**Scheme 3:**
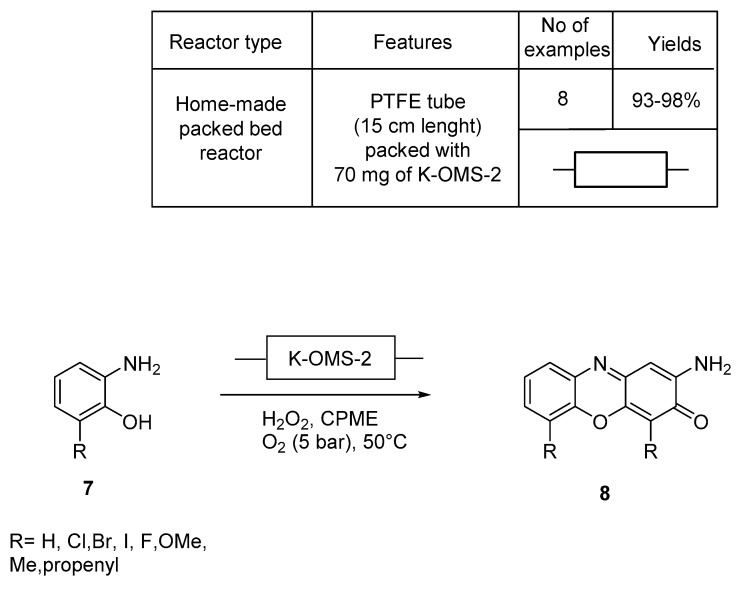
K+ form of octahedral molecular sievesK-OMS-2 catalyzed synthesis in flow of 2-aminophenoxazin-3-ones **8** through the C-H oxidation reaction.

The proposed synthesis employed substituted *o*-aminophenols **7,** that in the presence of a suitable oxidant and catalyst underwent C–H oxidative coupling to yield the desired 2-aminophenoxazin-3-ones **8** (Scheme 3).

The flow procedure herein discussed is a further improvement of the batch synthesis proposed by the same authors [25]. The catalyst of choice was the K+ form of octahedrall molecular sieves (K-OMS-2) first synthesized by Suib [26]. The structure possesses octahedral manganese oxides ions connected via edges and vertices with an average oxidation state of 3.8. The large surface area (up to 250 m^3^/g), robustness, and easy of synthesis make this catalyst an optimal candidate for oxidation processes. Careful optimization has been performed in order to choose the right combination of oxidant (O_2_/H_2_O_2_), amount of catalyst, and residence time. As a solvent, cyclopentylmethyl ether (CPME) was selected for its low toxicity profile and low tendency to form peroxides. In this tailor-made system, the catalyst was safely packed in a polytetrafluoroethylene PTFE tube (15 cm length), and only minimal leaching of the manganese catalyst was detected; the molecular oxygen line served as a driving force to move the reaction mixture throughout the reactor. A variety of 2-aminophenoxazin-3-ones **8** has been therefore synthesized with excellent yields (93–98%) and no need for further purification. The sustainability of the process was highlighted by E-factor values calculation and comparison with the batch protocol and other literature processes values. From this green metric, it was possible to evaluate the beneficial effect of the flow methodology that allowed obtaining E-factor values as low as 1.6 in flow, whereas values in the range of 19 were obtained for the corresponding batch procedures; literature procedures showed even higher E-factor values (68–368).

**Scheme 4:**
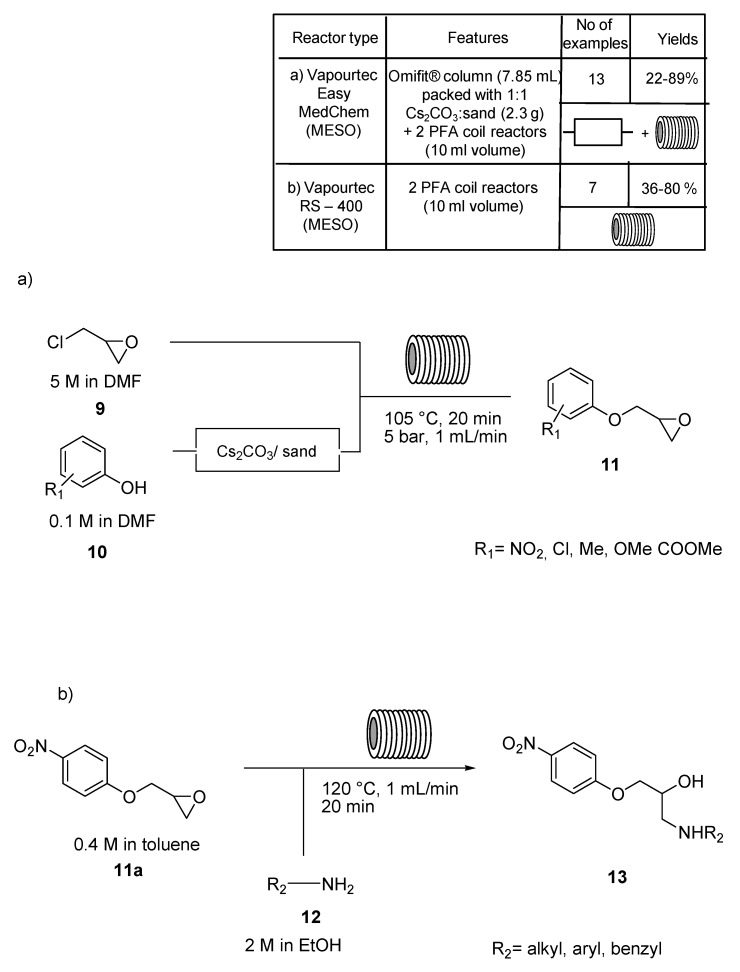
(**a**) Flow synthesis of epoxy ethers **11** from epichlorohydrin **9**. (**b**) Flow synthesis of *β*-amino alcohols **13** from amine mediated-epoxide ring-opening process.

*β*-Amino alcohols are often key intermediates for the synthesis of biologically-active molecules; they are typically synthesized by amine-mediated ring-opening of epoxides. Following this strategy, McCluskey and co-workers studied the reaction of epichlorohydrin **9** with phenols **10** to yield the corresponding epoxides **11** (Scheme 4a) [27].

The optimal reaction conditions were found by pumping a 0.1 M solution of phenol **10** in DMF through an Omnifit ^®^ column packed with a 1:1 mixture of Cs_2_CO_3_: acid-washed sand, to avoid the formation of the diol by-product, and then mixing such solution with epoxide **9** in DMF (5 M). The reactants were allowed to flow through two 10 mL PFA reactor coils at 105 °C for 20 min of residence time. A wide array of epoxides **11** were synthesized using this protocol, with yields ranging from acceptable to very good (22–89%). The epoxy derivative **11a** obtained by the reaction of epichlorohydrin and 4-nitrophenol was used as starting material to synthesize a library of *β*-aminophenols **13** through amine mediated epoxide ring-opening (Scheme 4b). A variety of primary, secondary, and tertiary amines **12** were used (5–7 equivalents) in flow to open epoxide **11a** with a good regiocontrol. Yields ranged from modest to very good (36–80%), with the most common by-products being the primary alcohol and bis-alkyl compounds.

**Scheme 5:**
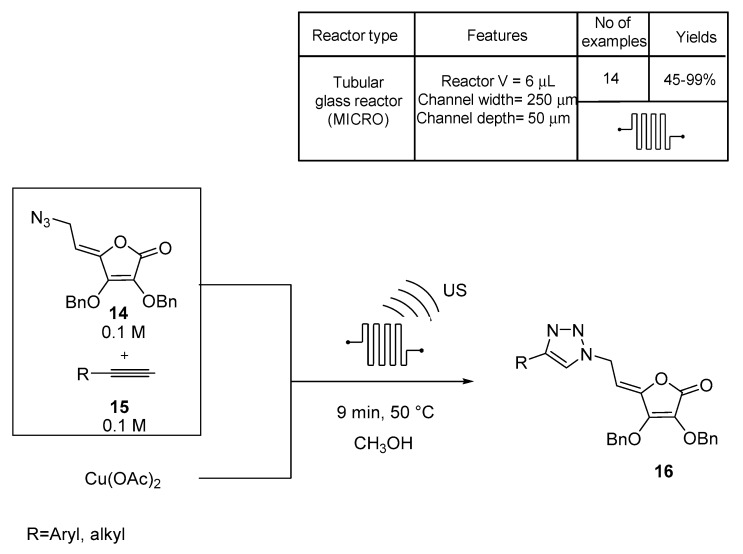
A combination of continuous flow and ultrasonic irradiation for the click reaction-based synthesis of new 1,2,3-triazolyl appended 4,5-unsaturated l-ascorbic acid derivatives.

Raić-Malić and co-workers published an interesting study on the flow-synthesis of a new series of molecules containing either the l-ascorbic acid (i.e., Vitamin C) and 1,2,3-triazole moieties [28]. The idea behind the work was to combine the well-known properties of Vitamin C with those of 1,2,3 triazoles and test such new molecules for biological properties. The authors first synthesized the azide derivative of l-ascorbic acid **14** to be employed in a click chemistry reaction with a wide array of alkynes **15** to form the 1,2,3-triazole unit (Scheme 5). Specifically, in a microfluidic system combined with ultrasound (US) irradiation, 1,2,3-triazolyl appended 4,5-unsaturated l-ascorbic acid derivatives **16** were synthesized through a copper-mediated 1,3 dipolar cycloaddition of C–6 azido l-ascorbic acid derivatives **14** and terminal alkynes **15**. The same transformation has also been performed in batch condition, but the flow methodology gave superior results either in terms of yields and of reaction times. In fact, for each of the 14 new products **16** the yield in flow resulted to be significantly better than that obtained in batch; furthermore, the optimal reaction time in flow resulted in being in the range of minutes whilst the reaction time in the batch was in the range of hours. Additionally, from the point of view of the safety profile, the flow protocol was again the preferred option; in fact, when extremely reactive azides are handled, it is of great advantage to able to let the reaction run inside proper equipment instead of performing traditional batch procedures [29].

## 3. *N*-Based Compound Libraries

**Figure 1:**
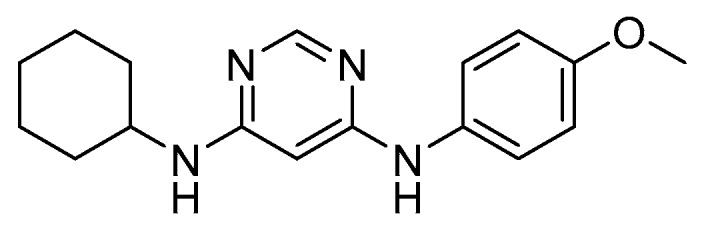
Structure of the cardiomyogenesis-inducing agent VUT-MK142.

**Scheme 6:**
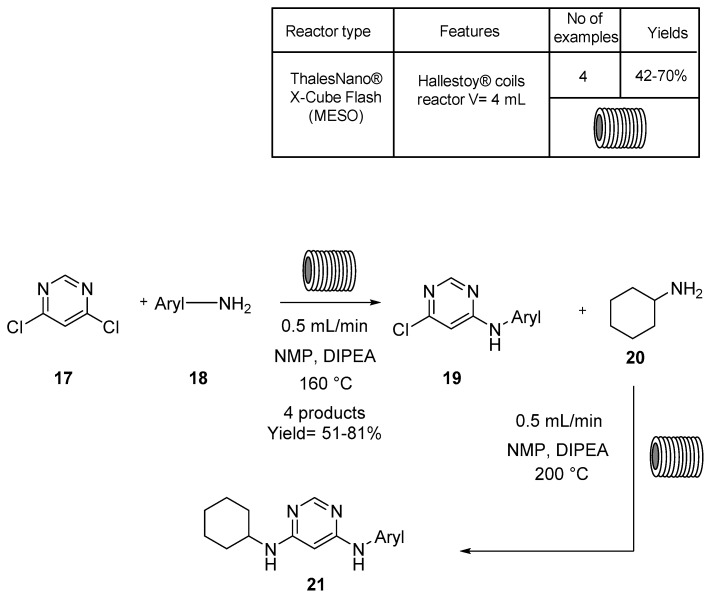
Two-step one flow synthesis of cardiomygenesis-inducing compounds.

4,6-Disubstituted pyrimidines are promising molecules to be employed in the field of artificial cell differentiation [30]. In particular, VUT-MK142 (Figure 1) has proven to be an efficient cardiomyogenesis-inducing agent. Schnürch and co-workers, therefore, proposed a new two-step one flow protocol for the synthesis of a small library of 4,6 substituted pyrimidines, as reported in Scheme 6 [31].

In the first step, nucleophilic substitution of 4,6-dichloropyrimidine **17** with aromatic amines **18,** occurred at 160 °C in a ThalesNano X-Cube Flash at 0.5 mL/min in NMP under base catalysis (DIPEA) to yield mono-substituted pyrimidines **19**. Such intermediates could be isolated and characterized, but for a more efficient process, they could also be directly mixed with cyclohexylamine **20** to perform the second step in the same reactor at 200 °C at 0.5 mL/min. Screening of the reaction conditions such as the concentration of the reactants and temperature were monitored using UHPLC analysis. In conclusion, a library of 4,6-disubstituted pyridines **21** was successfully synthesized using a flow system that allowed to obtain good yields (51–81% first step and 42–70% second step) with a residence time in the range of minutes.

Tricyclic-tetrahydroquinolines (TC-THQs) are of great interest as they display a wide array of biological properties. In fact, they are common scaffolds in numerous biologically active natural compounds as well as drugs [32]. Gioiello and co-workers have set up a flow system for the multicomponent synthesis of a library of TC-THQs with drug-like properties in order to test their biological activity [33].

**Scheme 7:**
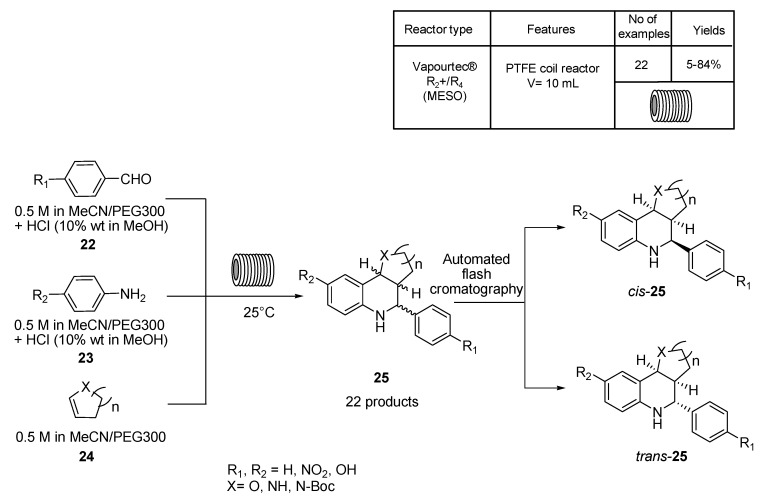
Flow synthesis of tetrahydroquinoline-based compound library.

The reaction of choice to build-up the molecular skeleton was the Pavarov cycloaddition in which p-substituted benzaldehydes **22**, anilines **23** and five- or six-member dienophiles **24** dissolved in MeCN/PEG300 (95:5 *v/v*) were combined together in a 10 mL PTFE reactor coil in the presence of HCl as catalyst to yield the desired tricyclic-tetrahydroquinolines **25** (Scheme 7).

The choice of using PEG300 as co-solvent made the purification procedure more complex, but, on the other hand, it was essential as it prevented the precipitation of the aniline chlorohydrates inside the system. Furthermore, the flow apparatus was equipped with an inline work-up system. Specifically, a stream of H_2_O, and Et_2_O was added to remove salts and PEG300. The products were purified by an automatic silica gel chromatography equipped with UV detector for fraction collection. This combined system allowed the synthesis of 22 products that have displayed biological activity in preliminary in vitro essays. As a continuation of this work, a study on the synthesis of tetracyclic quinolines, to be tested as ligands of the Pregnane X receptor (PXR), was performed by the same authors [34].

## 4. *N,S*-Based Compound Libraries

**Scheme 8:**
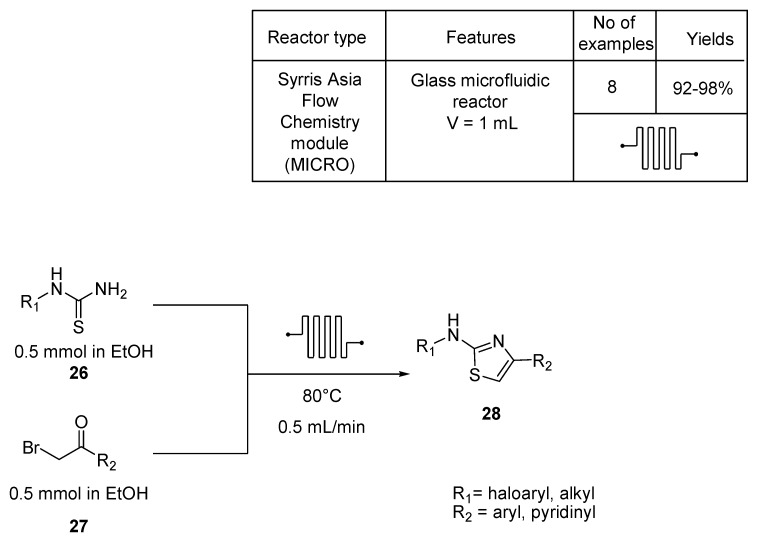
Microfluidic flow protocol for the synthesis of small molecules mimetic of the Humanin peptide.

A library of small molecules, namely 1-aminothiazole derivatives, has been synthesized in flow by John and co-workers in the framework of a wider project concerning Alzheimer’s disease (AD) [35]. In particular, the authors aimed to obtain small molecules that mimic the Humanin (HN) peptide interaction with the extracellular domain of the gp130 receptor in agonist fashion and ultimately protect neurons from NMDA-induced neurotoxicity [36]. In order to synthesize 1-aminothiazoles **28** the Hantzsch condensation reaction was chosen, therefore, a set of 1-(4-bromophenyl)thiourea derivatives **26** and 2-bromo-1-phenylethan-1-ones **27** were allowed to react in a microfluidic system at 80 °C without the aid of any catalyst (Scheme 8). It was possible to obtain eight small molecules in very good yields and suitable for gaining SAR insights. In particular, the 1-aminothiazole with R_1_ = 4-fluoroaryl and R_2_ = aryl resulted in being the best candidate for neuroprotective activity and, therefore, will be used for in vivo tests.

**Scheme 9:**
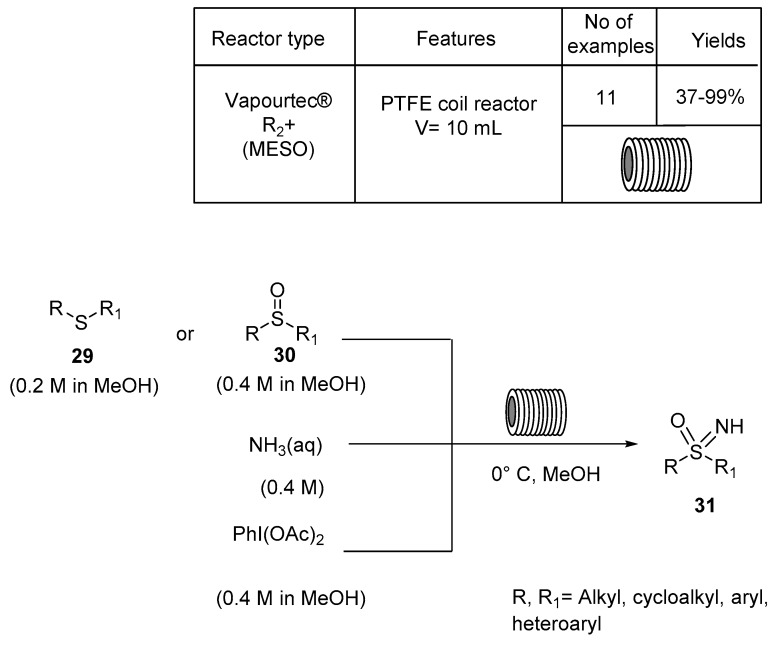
Flow synthesis of an NH-sulfoxamine library.

NH-Sulfoximines are an interesting class of compounds that, even though not very commonly found in the literature, are rapidly gaining the attention of the scientific community [37,38]. Such spike in interest is due to their use in the synthesis of sulfurated products, and in the drug discovery field, in fact, some sulfoximines-bearing compounds are promising pharmaceutically active molecules [39,40]. Luisi and co-workers set up a new convenient protocol for the synthesis in flow of sulfoximines starting from sulfides or sulfoxides [41]. The procedure is a modified and improved version of a batch procedure described by the same group [42]. The conversion of sulfides or sulfoxides occurred in the presence of an ammonia source and of phenyliodo diacetate (PhI(OAc)_2_). Careful evaluation of experimental parameters such as solvent, reactants stoichiometry, and concentrations and source of ammonia was performed in order to set up a flow system able to smoothly afford the desired NH-sulfoximines. All the optimization studies were performed using the commercial apparatus Vapourtec R2 system equipped with a 10 mL PTFE coil. Methanol was chosen as solvent as it proved to be ideal in the batch reaction, an aqueous solution of ammonia (28% *w/w*, 3 equiv.) was selected as N source. Other reagents proved to be effective but more difficult to handle. The amount of PhI(OAc)_2_ employed was lowered to 2 equivalents compared to 2.5 equiv. used in the batch procedure. When sulfides were used as starting material, the concentration was set to 0.2 M in order to avoid clogging of the reactor, but when sulfoxides were employed more concentrated solution was needed (0.4 M) for the reaction to occur. In the latter case syringe pumps were employed to feed the reactor. Finally, with the optimized conditions in hand, the authors were able to employ different sulfides **29** and sulfoxides **30** to yield 11 NH-sulfoximines **31** with yields ranging from 37 to 99% (Scheme 9).

## 5. Conclusions

Over the past two decades, continuous flow chemistry has proven to be an ideal methodology for running reactions that underperform in batch. Among the advantages of this technology are to be mentioned better control over heat and mass transfer, automation of operation, and a safer environment. These features are indeed of great interest in the drug discovery field, where the need for screening a wide array of compounds demands fast and reliable synthetic protocols. This review exemplified such a concept by discussing flow chemistry papers from recent literature. Regardless of the type of equipment used and of the reaction scale, this enabling technology showed its prominent role in the rapid and efficient assembly of compound libraries.
